# Supplementary material for: Personality predicts the propensity for social learning in a wild primate
Source: PeerJ. 2014 Mar 11;2:e283. doi: 10.7717/peerj.283 (PMC3961137; doi:10.7717/peerj.283)

Supplementary material for:

PERSONALITY PREDICTS THE PROPENSITY FOR SOCIAL LEARNING IN A WILD PRIMATE

Alecia J. Carter, Harry H. Marshall, Robert Heinsohn & Guy Cowlishaw

While we found no interaction between the presentation number and personality type that would suggest social learning (see main text), we did find that juvenile baboons showed greater responses to and improvement in the hidden food task with greater personal experience of the task. We assessed the evidence for social learning in different age classes by analysing the intensity of response to the stimulus in relation to the number of prior total presentations following the model specifications for $m3_{HF}$.

Supplementary table: Factors affecting social learning of the hidden food task when an interaction between the presentation number and age class is considered.

Experiment	Response	Term	β	S.E	*z*
Hidden food: total information	Response (levels 0-4)	Intercept	3.16	0.45	6.98
		Presentation number	-0.20	0.07	-2.72
		Age class: juvenile	0.24	0.45	0.54
		Boldness: shy	-1.15	0.30	-3.82
		Presentation number * Age class: juvenile	0.22	0.08	2.65

Supplementary figure: The predicted responses of baboons to the hidden food task with increasing personal experience of the task. Open circles and dotted lines show the predictions for juveniles, filled circles and solid lines indicate those of adults. Thick lines indicate bold baboons, and thin lines indicate shy baboons.

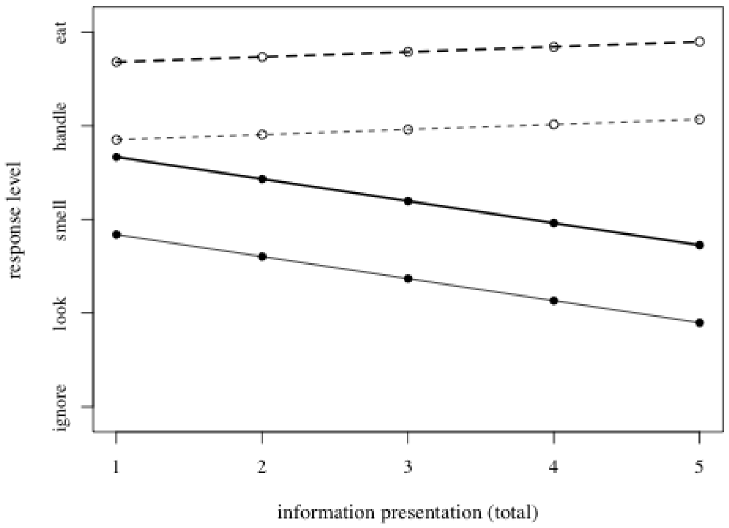

Supplement: File S1 — Factors affecting social learning of the hidden food task when an interaction between the presentation number and age class is considered. [file peerj-02-283-s002.pdf]
